# Age-independent benefits of postoperative rehabilitation during chemoradiotherapy on functional outcomes and survival in patients with glioblastoma

**DOI:** 10.1007/s11060-024-04785-1

**Published:** 2024-07-30

**Authors:** Keisuke Natsume, Akira Yoshida, Harutoshi Sakakima, Hajime Yonezawa, Kentaro Kawamura, Shintaro Akihiro, Ryosuke Hanaya, Megumi Shimodozono

**Affiliations:** 1 Department of Rehabilitation and Physical Medicine, Graduate School of Medical and Dental Sciences, Kagoshima University, Kagoshima, Japan; 2 Department of Physical Therapy, School of Health Sciences, Faculty of Medicine, Kagoshima University, Kagoshima, Japan; 3 Department of Neurosurgery, Graduate School of Medical and Dental Sciences, Kagoshima University, Kagoshima, Japan

**Keywords:** Glioblastoma, Older individuals, Rehabilitation, Activities of daily living, Survival

## Abstract

**Purpose:**

To investigate the impact of early and continuous postoperative inpatient rehabilitation during chemoradiotherapy on functional outcomes and overall survival (OS) in patients with glioblastoma (GBM), particularly in different age groups.

**Methods:**

This retrospective cohort study at a university hospital (2011–2016) included 75 of 119 consecutive patients newly diagnosed with GBM who underwent standardized treatment and postoperative rehabilitation. Patients were divided into older (≥ 65 years, *n* = 45) and younger (< 65 years, *n* = 30) groups, engaging in a 50-day rehabilitation program. We assessed rehabilitation progress, Barthel Index (BI), Brunnstrom Recovery Stage (BRS), adverse events, and OS. BI at discharge and survival were analyzed using multivariate and Cox regression models, respectively.

**Results:**

The mean age was 72.5 ± 6.3 and 52.4 ± 7.8 years in the older and younger groups, respectively. Both groups demonstrated significant improvements in BI and BRS. Despite more adverse events in the older group, no significant difference existed in median OS (older group: 18.7 months vs. younger group: 18.3 months, *p* = 0.87). Early walking training, reduced fatigue during chemoradiotherapy, and high Karnofsky Performance Status at admission significantly impacted the BI at discharge. Cox regression analysis identified the BI at discharge as a significant predictor of survival (hazard ratio [HR] 0.98, 95% confidence interval [CI] 0.97–0.99, *p* = 0.008).

**Conclusion:**

Integrated rehabilitation improves functional outcomes, and enhanced ADL at discharge is associated with improved survival outcomes in patients with GBM, regardless of age. This highlights the need for personalized rehabilitation in treatment protocols. Further prospective studies are warranted to confirm these findings.

**Supplementary Information:**

The online version contains supplementary material available at 10.1007/s11060-024-04785-1.

## Introduction

Glioblastoma (GBM) is the most prevalent primary malignant brain tumor, with a notably poor prognosis [1]. The median overall survival (OS) is 15.6 months [2], and the 5-year survival rate is 6.8% [3]. The incidence rates increase with age, peaking among those aged 75–84 years [3], where the median OS drops to 9.1 months, and the 5-year survival rate is only 5.3% for patients aged > 65 years [4].

The standard treatment for GBM patients aged < 70 years with good performance status includes maximal surgical resection followed by radiotherapy and chemotherapy using temozolomide (TMZ) [5, 6]. However, older patients often have limited treatment options due to poor prognosis and higher risk of side effects, resulting in their frequent exclusion from clinical trials. Although certain studies have indicated the effectiveness of standard treatments in older patients [7, 8] and others have proposed hypo-fractionated radiotherapy as a viable alternative [9, 10], these evaluations primarily focused on patients with a favorable performance status. However, the potential benefits of rehabilitation treatments in enhancing outcomes for older patients with GBM, who frequently have a poor performance status, remain insufficiently investigated.

Notably, some studies demonstrated that inpatient rehabilitation improved activities of daily living (ADL) in patients with brain tumors [11–14], similar to that in patients with stroke [15–17] and traumatic brain injury [18]. These studies included patients with various types of brain tumors. Few studies focused on the effectiveness of inpatient rehabilitation in patients with GBM [19–24]. Such interventions are promising in enhancing functional independence and potentially extending survival [19, 21, 24]. However, treatments used in conjunction with rehabilitation vary, and it is unclear which treatments ultimately affect outcomes. Despite these findings, the impact of rehabilitation on functional outcomes and survival, especially among older patients with GBM, remains underexplored.

This study aimed to investigate the impact of early postoperative and ongoing inpatient rehabilitation combined with standardized chemoradiotherapy on functional outcomes and OS in older (≥ 65 years) and younger (< 65 years) patients with GBM.

## Methods

### Patient selection process

This was a retrospective cohort study of consecutive patients newly diagnosed with GBM who were admitted to the neurosurgery department of a single university hospital between January 2011 and January 2016. The inclusion criteria were as follows: (1) patients aged ≥ 18 years and (2) diagnosed with histologically confirmed GBM. The exclusion criteria were as follows: (1) patients who exclusively underwent surgery (*n* = 5), received only radiotherapy (*n* = 5), or solely received TMZ after surgery (*n* = 3); (2) individuals who postoperatively underwent treatment involving TMZ concurrently with radiotherapy and bevacizumab (*n* = 20); (3) patients who solely received bevacizumab or palliative care postoperatively (*n* = 1 each); and (4) those who opted out of postoperative rehabilitation owing to personal or familial objections (*n* = 9). Inpatient rehabilitation was standard for all GBM patients undergoing surgery or chemoradiotherapy unless declined by the patient or their family. Of the initial 119 patients, 75 who postoperatively received TMZ and radiotherapy and underwent inpatient rehabilitation met the inclusion criteria. They were categorized into an older group (≥ 65 years, *n* = 45) and a younger group (< 65 years, *n* = 30). This study was conducted in accordance with the Strengthening the Reporting of Observational Studies in Epidemiology guidelines for cohort studies. It was approved by the Ethics Committee of Kagoshima University Graduate School of Medical and Dental Sciences (07/06/2016, no. 28–69 and 24/06/2016, no. 28–70) and followed the Declaration of Helsinki principles. Consent was indirectly confirmed through public disclosure on our official website.

### Treatment course

The operation was performed within 3 days after admission, followed by TMZ administration combined with radiotherapy, in accordance with the Stupp protocol. This comprised 75 mg/m^2^ TMZ daily for 6 weeks and concomitant radiotherapy (dose 40–60 Gy). Twenty-eight days after discharge, patients received adjuvant TMZ (dose 150–200 mg/m^2^) for a variable number of cycles, depending on side effects and clinical judgment. Additionally, bevacizumab was administered as part of the treatment regimen for selected patients based on their clinical condition and treatment response.

### Inpatient rehabilitation protocol

During the early postoperative period, the focus was placed on icreasing patients’ level of consciousness. This progression involved moving from bed rest to sitting, wheelchair mobility, and eventually walking training. For patients with moderate-to-severe motor paralysis, repetitive facilitation exercises were used [25], aiming for independent ambulation before chemoradiotherapy.

Managing fatigue while advancing rehabilitation is crucial during chemoradiotherapy. Our program focuses on light- to moderate-intensity aerobic exercises, such as walking and treadmill use, tailored to patients’ endurance and fatigue levels. For older patients with impaired consciousness, the program included cognitive exercises such as memory tasks and problem-solving activities. Due to the challenges of direct cognitive training, wheelchair mobility sessions were organized to allow patients to enjoy natural scenery and social interaction. Tablets were used to play familiar music and show historical visuals for enhancing reminiscence therapy. Occupational therapists curated engaging and soothing content to ensure emotional stability and drew positive responses from patients.

A multidisciplinary team, including physiatrists, leads a comprehensive approach. The program involves 20–40-min therapy sessions conducted 5 days a week for 6 weeks or more. This approach is dynamically adjusted based on the patient’s progress and response, covering physical rehabilitation and the psychological and social aspects of well-being.

### Outcome measures

The primary outcome of the study was focused on OS, which was defined as the duration from the date of operation to either the date of death or the last follow-up. 

The Secondary outcomes encompassed various aspects of functional recovery and treatment adherence. ADLs were evaluated using the Barthel Index (BI) at the initiation of postoperative rehabilitation and discharge, providing insights into patients’ independence levels. A total score of BI ranges from 0 to 100, with higher scores indicating increased levels of independence. Motor paralysis was assessed through the Brunnstrom Recovery Stage (BRS) by certified physiatrists, offering a comprehensive evaluation of motor function in the upper extremity, hand, and lower extremity. The BRS comprises six stages, with higher stages indicating improved motor function. Additionally, the study monitored rehabilitation progress, including the time to initiate rehabilitation, sitting training, and walking training, to gauge the timely delivery of rehabilitative care. Moreover, adherence to the prescribed rehabilitation regimen was evaluated to assess patient compliance and engagement in the treatment process. Adverse events were closely monitored throughout chemoradiotherapy, using the Common Terminology Criteria for Adverse Events (CTCAE) version 3.0 to ensure the safety and tolerability of treatment protocols. Additional details on these methodologies are available in the Supplementary Explanation of Assessment Methods (Online Resource 1).

### Patient characteristics

Patient characteristic data were collected from medical records. This information included age, sex, Karnofsky Performance Status (KPS) at admission and discharge, tumor location and hemisphere, extent of resection, time from operation to initiation of chemoradiotherapy, length of hospital stay, contents of radiotherapy and chemotherapy, and discharge destination.

### Statistical analyses

Statistical analyses employed the Student’s t-test or Mann–Whitney U test for continuous and ordinal variables, respectively, and the chi-square or Fisher’s exact tests for categorical variables. Analyzed variables included age, hospital stay, and timing of rehabilitation initiation and training outcomes. Multiple regression and Cox regression models identified predictors of discharge BI scores and OS. Kaplan–Meier curves with log-rank tests were used to compare survival distributions. Details on these methodologies are available in the Supplementary Explanation of Multivariate Analysis (Online Resource 2). Analyses were conducted using IBM SPSS Statistics software (version 26; IBM Corporation, Armonk, NY, USA).

## Results

### Patient characteristics

Patient characteristics are summarized in Table 1. The mean age difference between the older and younger groups was 22 years. No significant differences were noted between the groups in terms of KPS at admission and discharge, tumor location, extent of resection, radiotherapy dosage, adjuvant therapy, and length of hospital stay. Fewer patients were discharged in the older group than in the younger group (*p* < 0.01).

### Rehabilitation progress

Table 2 shows that there was no significant difference in the initiation times of rehabilitation, sitting, and walking training between the older and younger groups. Notably, the commencement times for these rehabilitation activities were closely aligned between both groups. The proportion of patients unable to start walking training was not significantly different, underscoring similar rehabilitation initiation rates across age groups.

### Motor paralysis and ADL

Both groups exhibited significant improvements in BI scores, increasing by 40–45 points, and advanced across BRS stages for the upper extremity, hand, and lower extremity, demonstrating consistent recovery in motor function and daily activities without significant differences between the groups (Table 2).

### Rehabilitation compliance rate

The compliance rate to the rehabilitation regimen was high and similar between the groups, highlighting effective engagement in prescribed activities (Table 2).

**Table 1:** Demographic and clinical characteristics of patients with glioblastoma by age group^a^

	Overall (*n* = 75)	≥ 65 years (*n* = 45)	< 65 years (*n* = 30)	*p*-value^b^
Age, mean (SD)	65.8 (13.4)	74.6 (5.4)	52.7 (10.8)	
Sex, n (%)				0.08
Female Male	31 (41.3) 44 (58.7)	22 (48.9) 23 (51.1)	9 (30.0) 21 (70.0)	
KPS at admission, median (IQR) KPS at discharge, median (IQR)	60 (50–70) 60 (40–60)	60 (50–60) 60 (35–60)	60 (50–70) 60 (50–60)	0.08 0.22
Tumor location, n (%)				0.45
Frontal Parietal Temporal Occipital Other	16 (21.3) 19 (25.4) 30 (40.0) 4 (5.3) 6 (8.0)	8 (17.8) 12 (26.6) 17 (37.8) 4 (8.9) 4 (8.9)	8 (26.7) 7 (23.3) 13 (43.3) 0 2 (6.7)	
Hemisphere, n (%)				0.16
Rt Lt Bilateral	37 (49.3) 31 (41.3) 7 (9.3)	22 (48.9) 21 (46.7) 2 (4.4)	15 (50) 10 (33.3) 5 (16.7)	
Extent of resection, n (%)				0.47
Gross total resection Near total resection Partial resection Biopsy	27 (36.0) 26 (34.7) 19 (25.3) 3 (4.0)	16 (35.5) 14 (31.1) 12 (26.7) 3 (6.7)	11 (36.7) 12 (40.0) 7 (23.3) 0	
Chemoradiotherapy, n (%)				0.69
TMZ + Conventional 60 Gy TMZ + Conventional 40 Gy TMZ + Conventional 40 Gy + cyberknife 20 Gy	31 (41.3) 6 (8.0) 38 (50.7)	20 (44.4) 4 (8.9) 21 (46.7)	11 (36.7) 2 (6.7) 17 (56.6)	
Mean (SD) days from surgery to CRT	16.1 (5.1)	16.7 (5.6)	15.3 (4.1)	0.23
Adjuvant therapy, n (%)				0.26
TMZ TMZ + Bevacizumab Best supportive care	20 (26.7) 44 (58.6) 11 (14.7)	14 (31.1) 23 (51.1) 8 (17.8)	6 (20) 21 (70) 3 (10)	
Length of hospital stay, mean (SD), days	62.2 (14.3)	63.2 (15.1)	60.7 (13.1)	0.46
Discharge destination, n (%)				0.01
Home Rehabilitation hospital Care facility	45 (60) 26 (35) 4 (5)	22 (49) 19 (42) 4 (9)	23 (76) 7 (24) 0	

^a^IQR, interquartile range; KPS, Karnofsky performance status; SD, standard deviation; TMZ, temozolomide; CRT, chemoradiotherapy

^b^The *p*-value indicates a comparison between the older and younger groups

**Table 2:** Comparison of rehabilitation outcome after operation in the older and younger groups^a^

	≥ 65 years (*n* = 45)	< 65 years (*n* = 30)	*p*-value
Early rehabilitation after surgery			
Starting rehabilitation (days) Starting sitting training (days) Starting walking training (days)	3.2 (2.3) 4.2 (2.4) 8.1 (6.4)	3.9 (3.0) 6.1 (5.1) 11.6 (14.6)	0.26 0.07 0.24
ADL (BI)			
T0 T1	30 (0–55)^b^ 75 (15–95)	35 (15–65)^b^ 75 (50–95)	0.23 0.23
Motor paralysis (BRS) Upper extremity			
T0 T1	4 (2–5)^b^ 5 (4–6)	4 (2–5)^c^ 6 (4–6)	0.88 0.53
Finger			
T0 T1	5 (2.5–5)^b^ 5 (4–6)	5 (3–5)^c^ 6 (4–6)	0.94 0.63
Lower extremity			
T0 T1	4 (2.3–5)^b^ 5 (4–6)	4 (2–5)^c^ 5 (3–6)	0.36 0.98
No motor paralysis n (%)			
T0 T1 Rehabilitation compliance rate n (%)	12 (26.7) 11 (24.4) 44 (98)	11 (36.7) 11 (36.7) 30 (100)	0.44 0.30 1.00

^a^ADL, activity of daily living; BI, Barthel Index; BRS, Brunnstrom Recovery Stage; T0, at the beginning of rehabilitation; T1, at the end of rehabilitation

Values are expressed as the median (interquartile range)

^b^This value indicates a significant difference between T0 and T1 (*p* < 0.01)

^c^This value indicates a significant difference between T0 and T1 (*p* < 0.05)

### Adverse events

Table 3 illustrates the adverse events observed during chemoradiotherapy. Regarding hematological toxicity, the older group exhibited a significantly higher incidence of leukopenia. Conversely, concerning non-hematological toxicity, fatigue, and cognitive dysfunction were notably more prevalent in the older group. Furthermore, when considering severe adverse events (CTCAE grade 3 or 4), the older group demonstrated elevated incidences of leukopenia and cognitive dysfunction. Notably, severe infection was not observed in either group. At admission, cognitive dysfunction corresponding to CTCAE grade 1 or 2 was observed in 33 (73.3%) patients in the older and 12 (40%) in the younger group. In comparison, grade 3 or 4 cognitive dysfunction was observed in two (4.4%) patients in the older group and none in the younger group (*p* = 0.002). One (2.2%) patient in the older and one (3.3%) in the younger group interrupted radiotherapy (*p* = 1.00), and three (6.7%) patients in the older and none (0%) in the younger group discontinued radiotherapy (*p* = 0.27). Seven (15.5%) patients in the older and four (13.3%) in the younger group interrupted TMZ (*p* = 1.00), and nine (20.0%) patients in the older and three (10.0%) in the younger group discontinued TMZ (*p* = 0.34).

### Predictors of the BI score at discharge

Three factors were significantly associated with the BI score at discharge: the duration until starting walking training post-operation (β=-0.324, 95% confidence interval [CI] -34.86 to -10.66, *p* < 0.001), fatigue during chemoradiotherapy (β=-0.382, 95% CI -32.47 to -11.79, *p* < 0.001), and KPS at admission (β = 0.289, 95% CI 0.27–1.04, *p* = 0.001). Meanwhile, age (*p* = 0.96), the extent of resection (*p* = 0.13), and cognitive function post-operation (*p* = 0.113) were not significant predictors. Detailed statistical methodologies and model verification have been presented in Supplementary Material: Validity and Robustness of Multiple Regression Analysis (Online Resource 3).

**Table 3:** Hematologic and non-hematologic adverse events during chemoradiotherapy

	≥ 65 years (*n* = 45)				< 65 years (*n* = 30)			*p*-value
	Grade 0	Grade 1 or 2	Grade 3 or 4		Grade 0	Grade 1 or 2	Grade 3 or 4	
Hematologic toxicity n (%)^a^								
Leukopenia Neutropenia Lymphocytopenia Thrombocytopenia Anemia	18 (40) 31 (68) 4 (9) 9 (20) 1 (2)	20 (44) 7 (16) 24 (53) 35 (77) 41 (91)	7 (16) 7 (16) 17 (38) 1 (2) 3 (6)		21(70) 19 (68) 8 (27) 8 (27) 5 (17)	9 (30) 6 (21) 12 (41) 22 (73) 25 (83)	0 3 (11) 9 (31) 0 0	0.01 0.86 0.17 0.33 0.08
Non-hematologic toxicity n (%)^a^								
Fatigue Fever Depression Cognitive dysfunction	21 (50) 27 (60) 42 (93) 4 (9)	21 (50) 16 (35) 3 (7) 32 (71)	0 2 (4) 0 9 (20)		23 (79) 24 (80) 30 (100) 5 (17)	6 (21) 5 (17) 0 25 (83)	0 1 (3) 0 0	0.01 0.09 0.15 0.03

^a^Data for 1–3 patients are missing for some toxicities in both groups

### OS

**Fig. 1:**
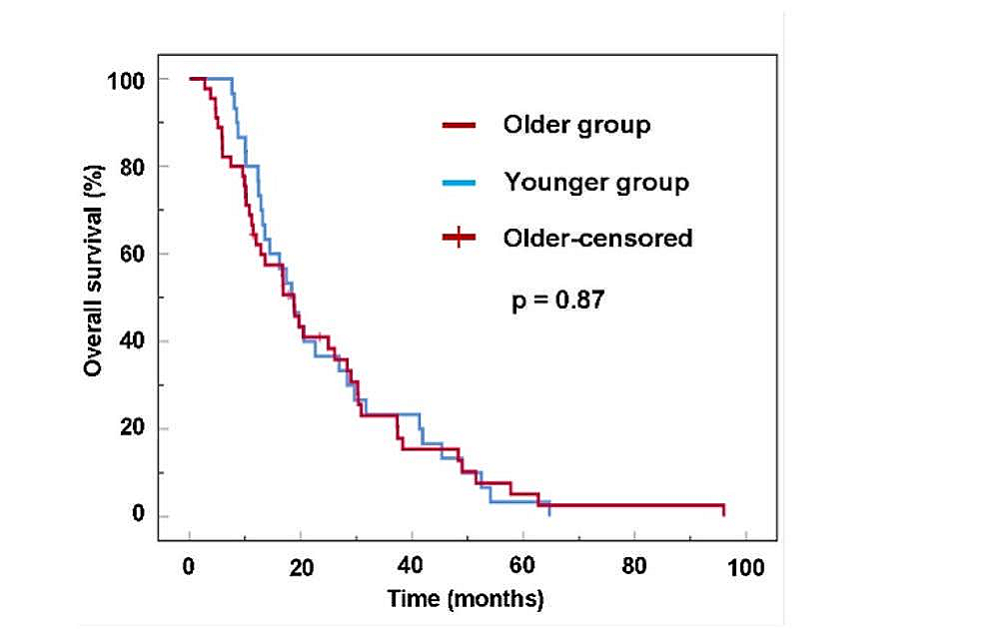
Kaplan–Meier estimates of overall survival in patients with glioblastoma who postoperatively received inpatient rehabilitation concomitant with chemoradiotherapy

The median OS was 18.7 (95% CI 15.7–21.7) months for the entire cohort, 18.7 (95% CI 15.0–22.5) months for the older group, and 18.3 (95% CI 13.7–23.0) months for the younger group (Fig. 1). Moreover, no significant between-group difference was observed (*p* = 0.87). Notably, 96.0% of patients died by the last follow-up date.

### Predictors of survival

The most significant predictor of better survival outcomes was the BI score at discharge. A wider extent of resection and less fatigue also showed a positive influence on survival, although this was not statistically significant. KPS at admission, severity of cognitive dysfunction after operation, and age were not significantly associated with survival (Table 4).

Subgroup analyses were conducted based on the change in BI scores before and after rehabilitation to evaluate its impact on survival. Subgroup analyses, stratified by age, sex, admission KPS, and extent of resection, revealed that rehabilitation led to significantly improved survival across most subgroups: ages < 65 (hazard ratio [HR] 0.95) and ≥ 65 (HR 0.97) years; males (HR 0.96); KPS < 70 (HR 0.96) and ≥ 70 (HR 0.97); and total/subtotal resections (HR 0.98) versus partial/biopsy (HR 0.97), although the effect in females was not statistically significant. (Supplementary Table S1 [Online Resource 4]).

**Table 4:** Cox regression analysis for predictors of survival^a, b^

	Adjusted HR	95% CI	*p*-value
Age Extent of resection KPS at admission Fatigue Severity of cognitive dysfunction after the operation BI at discharge	1.00 0.59 0.98 0.60 0.89 0.98	0.98–1.02 0.33–1.05 0.96–1.00 0.35–1.05 0.66–1.20 0.97–0.99	0.84 0.07 0.12 0.07 0.46 0.008

^a^BI, Barthel Index; KPS, Karnofsky performance score; HR, hazard ratio; CI, confidence interval

^b^Model Fit (chi-square) = 17.21, df (degrees of freedom) = 6, p (model) = 0.009

## Discussion

The key finding of our study was the improvement in ADL among both older and younger patients with GBM following postoperative inpatient rehabilitation combined with chemoradiotherapy. Furthermore, despite a mean age difference of 22 years between the groups, there was no significant difference in median OS, challenging the conventional view that age decisively affects outcomes in patients with GBM.

Studies have indicated that inpatient rehabilitation postoperatively enhances ADL among newly diagnosed GBM patients [19–23]. These studies employed the functional independence measure (FIM) to assess ADL. In comparison, we assessed ADL using BI because of its simplicity and correlation with KPS [26]. Prodinger et al. established score equivalence between the FIM motor and BI scores [27]. Based on their transformation table, these postoperative ADL scores align closely with those reported in a previous study [27]. However, ADL scores at discharge appear to be notably improved compared to prior findings.

Our study initiated rehabilitation treatment approximately 3 days postoperatively, significantly earlier than the 6–14 days reported in previous research [19–23]. This timely intervention, coupled with the sustained rehabilitation during chemoradiotherapy spanning approximately 50 days here—contrasting with the shorter duration of 11–28 days in prior research [19–23]—played a pivotal role in the notable improvement in ADL at discharge.

Standard GBM treatment entails postoperative chemoradiotherapy, which is less common in older patients due to increased risks of side effects [4]. Notably, grade 3 or 4 hematological toxicity occurs in 10–35% of older patients undergoing chemoradiotherapy [7, 28]. In our study, leukopenia was more prevalent in the older group, although severe infections were not observed. The absence of significant differences in treatment interruption or discontinuation rates between age groups, combined with high rehabilitation compliance, indicates the feasibility and importance of structured rehabilitation programs for enhancing ADL outcomes in patients with GBM.

Regression analysis revealed that early postoperative walking, reduced fatigue during chemoradiotherapy, and high KPS at admission significantly affected BI scores at discharge, with early walking being critical for ADL. Fatigue, related to sleep and emotional issues [29, 30], lowers motivation and ADL, impacting the quality of life. Poor performance status predicted fatigue severity [31], showing its relation to ADL. Despite higher levels of fatigue observed in older individuals, severe fatigue and ADL outcomes remained consistent across groups, indicating that rehabilitation effectively mitigates fatigue and enhances ADL, particularly in older patients.

Older age is associated with poor prognosis in patients with GBM [32]. Studies have shown that older patients tend to have shorter survival after postoperative chemoradiotherapy [33], with a marked difference in OS between those aged ≥ 65 years and those aged < 65 years [28]. However, in our study, despite expectations based on age and performance status indicators, we found no significant difference in OS between older and younger patients, challenging the conventional understanding of age as a determinant of survival in patients with GBM.

While the O-6-methylguanine-DNA methyltransferase promoter methylation status, strongly associated with prolonged survival in TMZ-treated GBM patients [8–10, 34], was not analyzed in our study, the existing literature suggests that its status remains age-independent and stable [35]. Therefore, its minimal impact on the observed OS differences between groups can be inferred.

Cox regression analysis revealed BI at discharge as a key survival predictor for GBM patients on postoperative rehabilitation integrated with chemoradiotherapy, surpassing traditional factors such as resection extent and age. This analysis underscores the significant impact of continuous rehabilitation from early postoperative stages to the end of chemoradiotherapy on survival. It suggests that combining rehabilitation with standard treatments aids functional recovery and potentially extends survival. These results support the inclusion of rehabilitation in GBM treatment protocols, advocating a holistic approach that emphasizes functional improvement.

Tang et al. showed that FIM gain in rehabilitation is age-independent and linked to extended OS [19]. In contrast, Roberts et al. identified older age, limited resection, and absence of the Stupp regimen as factors increasing mortality risk [21]. Our findings align with that of Tang et al., indicating that age does not influence ADL improvement and OS. A key difference between our study and Roberts et al.’s is the rehabilitation duration and treatment; all our patients received chemoradiotherapy, with a continuous 50-day rehabilitation period, compared with only 75% of patients in Roberts et al.’s study receiving chemoradiotherapy, with an average 13.2-day rehabilitation period. These differences may have influenced mortality rate variations among older patients. Our findings suggest that extended rehabilitation during chemoradiotherapy may be crucial for older patients with GBM. By focusing exclusively on patients with GBM receiving standardized treatment and directly comparing ADL outcomes between the older and younger groups, we highlight inpatient rehabilitation’s potential to improve ADL outcomes equally across ages. Moreover, we have previously mentioned the possibility that ADL improvement through inpatient rehabilitation can lead to extended OS in patients with GBM [24], reinforcing the importance of incorporating rehabilitation into the treatment regimen for patients with GBM across all age groups.

Cognitive dysfunction was significantly higher in the older group. Mild cognitive dysfunction was substantially more frequent in older individuals at admission, and grade 3 or 4 cognitive dysfunction was observed only in the older group (rate 20%) during chemoradiotherapy. A higher incidence of cognitive dysfunction in older patients has been reported previously [8, 28]. Therefore, cognitive dysfunction might be associated with vulnerability to chemoradiotherapy and aging. Cognitive dysfunction is expected to be linked to lower ADL and quality of life; thus, managing cognitive dysfunction is vital for older patients.

Additionally, while fatigue and cognitive dysfunction, significant predictors of survival in high-grade glioma [31, 36, 37], were more common in older individuals, these factors did not alter the outcome disparities in our study. Notably, discharge BI and KPS scores, along with OS, showed no significant variation across age groups. This underscores the importance of targeted rehabilitation to enhance ADL. It highlights its critical role in extending survival among older patients with GBM with compromised performance status beyond the effects of fatigue or cognitive dysfunction.

Our research underscores the substantial advantages of incorporating rehabilitation into conventional treatment protocols, particularly for older adults. This integration enhances survival rates and functional outcomes. Detailed subgroup analysis revealed that these improvements were consistent across various patient demographics, emphasizing the importance of developing personalized rehabilitation plans for each distinct patient group.

This study has certain limitations. As an observational study, it could not establish causality. The absence of a control group means effects attributed to rehabilitation may be confounded by other interventions. Additionally, the small sample size and single facility location limit the generalizability of the results. Future randomized controlled trials and multicenter studies should validate and improve the generalizability of rehabilitation effects. Prospective studies should also explore the impact on quality of life, especially cognitive functions and fatigue.

## Conclusion

Our study underscores the significant benefits of integrating early inpatient rehabilitation with chemoradiotherapy for GBM patients, leading to enhanced survival and functional outcomes, irrespective of age. This research holds promise in transforming the management of older patients with GBM, providing them with opportunities for prolonged survival and enhanced functional independence. Given the retrospective nature of this study and the small sample size, conducting future prospective, controlled studies to solidify these conclusions and explore rehabilitation’s impact on quality of life and long-term outcomes is imperative.

## Electronic supplementary material

Below is the link to the electronic supplementary material.

Supplementary Material 1

Supplementary Material 2

Supplementary Material 3

Supplementary Material 4

## Data Availability

No datasets were generated or analysed during the current study.
