# Supplementary material for: Age-independent benefits of postoperative rehabilitation during chemoradiotherapy on functional outcomes and survival in patients with glioblastoma
Source: J Neurooncol. 2024 Jul 30;170(1):129–37. doi: 10.1007/s11060-024-04785-1 (PMC11447139; doi:10.1007/s11060-024-04785-1)
Supplement: Supplementary file 1 — Supplementary Material 1 [file 11060_2024_4785_MOESM1_ESM.docx]

**Online Resource 1**

Article title: Age-independent Benefits of Postoperative Rehabilitation during Chemoradiotherapy on Functional Outcomes and Survival in Patients with Glioblastoma

Journal name: *Journal of Neuro-Oncology*

Author names: Keisuke Natsume^1,2^, Akira Yoshida^1^, Harutoshi Sakakima^2^, Hajime Yonezawa^3^, Kentaro Kawamura^1^, Shintaro Akihiro^1,2^, Ryosuke Hanaya^3^, Megumi Shimodozono^1^

Affiliations:

^1^Department of Rehabilitation and Physical Medicine, Graduate School of Medical and Dental Sciences, Kagoshima University, Kagoshima, Japan

^2^Department of Physical Therapy, School of Health Sciences, Faculty of Medicine, Kagoshima University, Kagoshima, Japan

^3^Department of Neurosurgery, Graduate School of Medical and Dental Sciences, Kagoshima University, Kagoshima, Japan

Corresponding author: Akira Yoshida, MD, PhD, Department of Rehabilitation and Physical Medicine, Kagoshima University Graduate School of Medical and Dental Sciences, 8-35-1 Sakuragaoka, Kagoshima 890-8520, Japan

Phone: +81-99-275-5339

Fax: +81-99-275-1273

E-mail: akiray@m.kufm.kagoshima-u.ac.jp

**Supplementary Explanation of Assessment Methods**

In this study, various assessment tools were used to evaluate the functional outcomes and complications associated with postoperative rehabilitation and chemoradiotherapy in patients with glioblastoma. These tools include the Barthel Index (BI), Brunnstrom Recovery Stage (BRS), and the Common Terminology Criteria for Adverse Events (CTCAE).

**Barthel Index (BI)**

The BI is a widely used measure of functional independence in activities of daily living (ADLs), with a total score ranging from 0 to 100. Higher scores indicate increased level of independence [S1] [S2]. The score ranges are as follows:

Total dependence (BI ≤ 40): Indicates complete dependence on others.

Severe dependence (BI ≤ 60): Indicates a transition from complete dependence to assisted independence.

Independence (BI ≥ 85): Indicates independence with minimal assistance, suitable for community living.

The specific items evaluated using BI include:

Feeding:

10 = Independent. The patient can feed themselves.

5 = Requires some help.

Moving from wheelchair to bed and return:

15 = Independent in all phases.

10 = Needs some help or supervision.

5 = Requires considerable help.

Personal toilet:

5 = Can wash face, comb hair, clean teeth, and shave independently.

Getting on and off the toilet:

10 = Can handle clothes, get on/off the toilet, and use toilet paper independently.

5 = Needs some help.

Bathing self:

5 = Can bathe independently.

Walking on a level surface (or propelling a wheelchair if unable to walk):

15 = Can walk 50 yards independently.

10 = Needs some help or supervision.

5 = Can propel wheelchair independently.

Ascending and descending stairs:

10 = Can go up and down a flight of stairs independently.

5 = Needs help or supervision.

Dressing and undressing:

10 = Can dress and undress independently.

5 = Needs some help.

Controlling bowels:

10 = Complete bowel control.

5 = Occasional accidents or needs help.

Controlling bladder:

10 = Complete bladder control.

5 = Occasional accidents or needs help.

This comprehensive evaluation allows healthcare providers to assess the level of assistance a patient requires and track their progress over time.

**Brunnstrom Recovery Stage (BRS)**

The BRS is a widely used scale for evaluating motor paralysis in the central nervous system. It provides a comprehensive assessment of motor function in the upper extremity, hand, and lower extremity [S3]. The BRS comprises six stages, with higher stages indicating better motor function. The details of each stage are as follows:

Stage 1: Flaccidity; no voluntary movement.

Stage 2: Spasticity begins; minimal voluntary movement.

Stage 3: Increased spasticity; voluntary movement within synergy patterns.

Stage 4: Decreased spasticity; some movements out of synergy patterns.

Stage 5: Further decrease in spasticity; more complex movements.

Stage 6: Spasticity disappears; individual joint movements and coordination near normal.

**Common Terminology Criteria for Adverse Events (CTCAE)**

The CTCAE is a standardized classification and severity grading scale for adverse events in clinical trials, ranging from Grade 0 to Grade 5. This ensures the safety and tolerability of treatment protocols.

The CTCAE version 3.0 includes the following grades:

Grade 0: No symptoms.

Grade 1: Mild; asymptomatic or mild symptoms; clinical or diagnostic observations only; intervention not indicated.

Grade 2: Moderate; minimal local or noninvasive intervention indicated; limiting age-appropriate instrumental ADL.

Grade 3: Severe or medically significant but not immediately life-threatening; hospitalization or prolongation of hospitalization indicated; disabling; limiting self-care ADL.

Grade 4: Life-threatening consequences; urgent intervention indicated.

Grade 5: Death related to an adverse event.

These measures were administered by trained physical therapists or occupational therapists for the BI and by certified rehabilitation physicians for the BRS. Adverse events were assessed and graded by the clinical team according to the CTCAE version 3.0 criteria.

References:

[S1] Mahoney FI, Barthel DW (1965) Functional evaluation: the Barthel Index. Md State Med J 14:61-65. https://pubmed.ncbi.nlm.nih.gov/14258950/

[S2] Quinn TJ, Langhorne P, Stott DJ (2011) Barthel Index for stroke trials: development, properties, and application. Stroke 42:1146-1151. https://doi.org/10.1161/STROKEAHA.110.598540

[S3] Brunnstrom S (1966) Motor testing procedures in hemiplegia: based on sequential recovery stages. Phys Ther 46:357-375. https://doi.org/10.1093/ptj/46.4.357
