# Supplementary material for: Age-independent benefits of postoperative rehabilitation during chemoradiotherapy on functional outcomes and survival in patients with glioblastoma
Source: J Neurooncol. 2024 Jul 30;170(1):129–37. doi: 10.1007/s11060-024-04785-1 (PMC11447139; doi:10.1007/s11060-024-04785-1)
Supplement: Supplementary file 2 — Supplementary Material 2 [file 11060_2024_4785_MOESM2_ESM.docx]

**Online Resource 2**

Article title: Age-independent Benefits of Postoperative Rehabilitation during Chemoradiotherapy on Functional Outcomes and Survival in Patients with Glioblastoma

Journal name: Journal of Neuro-Oncology

Author names: Keisuke Natsume^1,2^, Akira Yoshida^1^, Harutoshi Sakakima^2^, Hajime Yonezawa^3^, Kentaro Kawamura^1^, Shintaro Akihiro^1,2^, Ryosuke Hanaya^3^, Megumi Shimodozono^1^

Affiliations:

^1^Department of Rehabilitation and Physical Medicine, Graduate School of Medical and Dental Sciences, Kagoshima University, Kagoshima, Japan

^2^Department of Physical Therapy, School of Health Sciences, Faculty of Medicine, Kagoshima University, Kagoshima, Japan

^3^Department of Neurosurgery, Graduate School of Medical and Dental Sciences, Kagoshima University, Kagoshima, Japan

Corresponding author: Akira Yoshida, MD, PhD, Department of Rehabilitation and Physical Medicine, Kagoshima University Graduate School of Medical and Dental Sciences, 8-35-1 Sakuragaoka, Kagoshima 890-8520, Japan

Phone: +81-99-275-5339

Fax: +81-99-275-1273

E-mail: akiray@m.kufm.kagoshima-u.ac.jp

**Supplementary Explanation of Multivariate Analysis**

In this study, multivariate analyses were conducted using both multiple regression and Cox proportional hazards models employing the forced entry method. This approach ensures a comprehensive evaluation of all variables selected based on their clinical relevance. These methodologies comprehensively evaluate the factors influencing discharge Barthel Index (BI) scores and overall survival (OS) in patients with glioblastoma.

**Independent variables for multiple regression analysis**

The independent variables used in the multiple regression analysis and their corresponding scales are as follows:

1 Extent of Resection: Total/subtotal vs. partial resection/biopsy (Categorical scale)

2 Karnofsky Performance Status at Admission: Rated from 0 to 100 (Ordinal scale)

3 Age: Treated as a continuous variable (Continuous scale)

4 Fatigue during Chemoradiotherapy: Assessed according to CTCAE (Ordinal scale)

5 Postoperative Cognitive Dysfunction: Assessed according to CTCAE (Ordinal scale)

6 Duration Until Starting Walking Training After Operation: Within 8 days vs. after 9 days (Categorical scale)

**Independent variables for Cox proportional hazards analysi**s

The independent variables used in the Cox proportional hazards analysis and their corresponding scales are as follows:

1 Extent of Resection: Total/subtotal vs. partial resection/biopsy (Categorical scale)

2 Karnofsky Performance Status at Admission: Rated from 0 to 100 (Ordinal scale)

3 Age: Treated as a continuous variable (Continuous scale)

4 Fatigue during Chemoradiotherapy: Assessed according to CTCAE (Ordinal scale)

5 Postoperative Cognitive Dysfunction: Assessed according to CTCAE (Ordinal scale)

6 BI at Discharge: Assessed at discharge (Ordinal scale)

**Selection of independent variables**

Variables were chosen based on clinical relevance and prior research findings [7, 8, 24, 28, 31, 32, 36, 37]. Both older and younger groups underwent rehabilitation concurrent with treatment, showing significant improvements in activities of daily living (ADL) at discharge, with no significant differences between the groups. Follow-up assessments revealed no differences in OS. We hypothesized that ADL abilities at discharge might be associated with survival. Including the BI at discharge as an independent variable in our Cox proportional hazards model allows for a detailed analysis of how ADL improvements influence survival duration. This study addresses a gap in the existing literature regarding the impact of functional status at discharge on survival, aiming to enrich knowledge in this area.

Logistic regression and Cox regression models adhered to the commonly recommended rule of 10 events per predictor variable. This ensures reliability and statistical rigor, minimizing bias and variability in the results. Vittinghoff & McCulloch (2007) suggest that reliability can be maintained even with fewer than 10 events per predictor variable under suitable conditions. Our study exceeded this standard, providing a robust foundation for evaluating causal relationships [S1].

**Reference**

[S1] Vittinghoff E, McCulloch CE (2007) Relaxing the rule of ten events per variable in logistic and Cox regression. Am J Epidemiol 165:710-718. https://doi.org/10.1093/aje/kwk052.
