# Supplementary material for: Age-independent benefits of postoperative rehabilitation during chemoradiotherapy on functional outcomes and survival in patients with glioblastoma
Source: J Neurooncol. 2024 Jul 30;170(1):129–37. doi: 10.1007/s11060-024-04785-1 (PMC11447139; doi:10.1007/s11060-024-04785-1)
Supplement: Supplementary file 3 — Supplementary Material 3 [file 11060_2024_4785_MOESM3_ESM.docx]

**Online Resource 3**

Article title: Age-independent Benefits of Postoperative Rehabilitation during Chemoradiotherapy on Functional Outcomes and Survival in Patients with Glioblastoma

Journal name: Journal of Neuro-Oncology

Author names: Keisuke Natsume^1,2^, Akira Yoshida^1^, Harutoshi Sakakima^2^, Hajime Yonezawa^3^, Kentaro Kawamura^1^, Shintaro Akihiro^1,2^, Ryosuke Hanaya^3^, Megumi Shimodozono^1^

Affiliations:

^1^Department of Rehabilitation and Physical Medicine, Graduate School of Medical and Dental Sciences, Kagoshima University, Kagoshima, Japan

^2^Department of Physical Therapy, School of Health Sciences, Faculty of Medicine, Kagoshima University, Kagoshima, Japan

^3^Department of Neurosurgery, Graduate School of Medical and Dental Sciences, Kagoshima University, Kagoshima, Japan

Corresponding author: Akira Yoshida, MD, PhD, Department of Rehabilitation and Physical Medicine, Kagoshima University Graduate School of Medical and Dental Sciences, 8-35-1 Sakuragaoka, Kagoshima 890-8520, Japan.

Phone: +81-99-275-5339

Fax: +81-99-275-1273

E-mail: akiray@m.kufm.kagoshima-u.ac.jp

**Supplementary Material**

**Validity and Robustness of Multiple Regression Analysis**

**Model Description**

We conducted a multiple regression analysis to identify predictors of the Barthel Index (BI) score at discharge. This analysis forcibly entered all predictive variables into the model. Independent variables included the duration until starting walking training post-operation, fatigue during chemoradiotherapy, Karnofsky Performance Status at admission, age, extent of resection, and cognitive function post-surgery.

**Model Construction Method**

We employed the forced entry method, in which all predictors were included in the model simultaneously. This approach allows each predictor's impact on the BI score at discharge to be evaluated independently while controlling for other variables. This method was chosen based on predefined theories or hypotheses, aiming to clarify answers to our research questions by including variables of specific clinical or biological importance.

**Assumption Verification**

Linearity: The linearity between the dependent and independent variables was assessed using scatter plots.

Normality: The normality of residuals was checked using the Shapiro–Wilk test, which showed no significant deviation from normality (p > 0.05).

Homoscedasticity: Constant variance of residuals was confirmed through visual inspection of residual plots.

Independence: The Durbin–Watson statistic was 1.793, indicating no significant autocorrelation in the residuals.

Multicollinearity: The variance inflation factor values were below 5 for all predictors, indicating that multicollinearity was not a concern in our model.

**Model Fit**

The adjusted R-squared was 0.577, explaining approximately 57.7% of the variance in the BI score at discharge. The model was statistically significant overall (F [6, 64] = 16.895, p < 0.001), indicating it is an effective predictive model.

**Statistical Power and Sample Size**

The sample size of 71 provided sufficient power (80%) to detect medium to large effects at a significance level of 0.05, as calculated using post-hoc power analysis.

**Potential Limitations**

While this analysis provides robust evidence for the identified predictors, we acknowledge the potential presence of unmeasured confounding variables that could affect the BI score, and the observational nature of the study may limit causal inferences.

**Conclusion**

The conducted statistical analyses adhere to standard practices, providing valid and reliable results. Future studies might consider a larger sample size or a prospective design to validate and extend these findings.
