# Supplementary material for: Age-independent benefits of postoperative rehabilitation during chemoradiotherapy on functional outcomes and survival in patients with glioblastoma
Source: J Neurooncol. 2024 Jul 30;170(1):129–37. doi: 10.1007/s11060-024-04785-1 (PMC11447139; doi:10.1007/s11060-024-04785-1)
Supplement: Supplementary file 4 — Supplementary Material 4 [file 11060_2024_4785_MOESM4_ESM.docx]

**Online Resource 4**

Article title: Age-independent Benefits of Postoperative Rehabilitation during Chemoradiotherapy on Functional Outcomes and Survival in Patients with Glioblastoma

Journal name: Journal of Neuro-Oncology

Author names: Keisuke Natsume^1,2^, Akira Yoshida^1^, Harutoshi Sakakima^2^, Hajime Yonezawa^3^, Kentaro Kawamura^1^, Shintaro Akihiro^1,2^, Ryosuke Hanaya^3^, Megumi Shimodozono^1^

Affiliations:

^1^Department of Rehabilitation and Physical Medicine, Graduate School of Medical and Dental Sciences, Kagoshima University, Kagoshima, Japan

^2^Department of Physical Therapy, School of Health Sciences, Faculty of Medicine, Kagoshima University, Kagoshima, Japan

^3^Department of Neurosurgery, Graduate School of Medical and Dental Sciences, Kagoshima University, Kagoshima, Japan

Corresponding author: Akira Yoshida, MD, PhD, Department of Rehabilitation and Physical Medicine, Kagoshima University Graduate School of Medical and Dental Sciences, 8-35-1 Sakuragaoka, Kagoshima 890-8520, Japan.

Phone: +81-99-275-5339

Fax: +81-99-275-1273

E-mail: akiray@m.kufm.kagoshima-u.ac.jp

**Supplementary Table S1**

**Subgroup Analysis of the Impact of Rehabilitation on Survival**

**Table S1** Subgroup analysis of the impact of rehabilitation on survival

|  | Adjusted HR | 95% CI | *p*-value |
| --- | --- | --- | --- |
| Age  < 65 years  ≥ 65 years  Sex  Male  Female  KPS at admission  < 70  ≥ 70  Extent of resection  Total and subtotal  Partial and biopsy | 0.95  0.97  0.96  0.98  0.96  0.97  0.98  0.97 | 0.93-0.98  0.96-0.99  0.95-0.98  0.96-1.00  0.95-0.98  0.95-0.99  0.96-0.99  0.95-0.98 | 0.001  0.0002  <0.0001  0.132  0.008  <0.0001  0.009  0.001 |

KPS, Karnofsky performance status; HR, hazard ratio; CI, confidence interval.

This supplementary table contains detailed Cox proportional hazards models analyzing the change in BI score before and after rehabilitation, adjusted for age, admission KPS, the extent of resection, and sex.

Variables: age (<65 years, ≥65 years), sex (male, female), admission KPS (<70, ≥70), and extent of resection (total/subtotal, partial/biopsy)
